# Precisely dating the Frasnian–Famennian boundary: implications for the cause of the Late Devonian mass extinction

**DOI:** 10.1038/s41598-018-27847-7

**Published:** 2018-06-22

**Authors:** L. M. E. Percival, J. H. F. L. Davies, U. Schaltegger, D. De Vleeschouwer, A.-C. Da Silva, K. B. Föllmi

**Affiliations:** 1 Institut des sciences de la Terre, Géopolis, Université de Lausanne, 1015 Lausanne, Switzerland; 2 Département des sciences de la Terre, Université de Genève, 1205 Genève, Switzerland; 3 MARUM—Center for Marine Environmental Sciences, University of Bremen, Leobenerstraße, 28359 Bremen, Germany; 4 Sedimentary Petrology Laboratory, Liège University, Sart Tilman B20, Allée du Six Août 12, 4000 Liège, Belgium; 5 Paleomagnetic Laboratory, Utrecht University, Budapestlaan 17, 3584 CD Utrecht, The Netherlands

## Abstract

The Frasnian–Famennian boundary records one of the most catastrophic mass extinctions of the Phanerozoic Eon. Several possible causes for this extinction have been suggested, including extra-terrestrial impacts and large-scale volcanism. However, linking the extinction with these potential causes is hindered by the lack of precise dating of either the extinction or volcanic/impact events. In this study, a bentonite layer in uppermost-Frasnian sediments from Steinbruch Schmidt (Germany) is re-analysed using CA-ID-TIMS U-Pb zircon geochronology in order to constrain the date of the Frasnian–Famennian extinction. A new age of 372.36 ± 0.053 Ma is determined for this bentonite, confirming a date no older than 372.4 Ma for the Frasnian–Famennian boundary, which can be further constrained to 371.93–371.78 Ma using a pre-existing Late Devonian age model. This age is consistent with previous dates, but is significantly more precise. When compared with published ages of the Siljan impact crater and basalts produced by large-scale volcanism, there is no apparent correlation between the extinction and either phenomenon, not clearly supporting them as a direct cause for the Frasnian–Famennian event. This result highlights an urgent need for further Late Devonian geochronological and chemostratigraphic work to better understand the cause(s) of this extinction.

## Introduction

**Figure 1:**
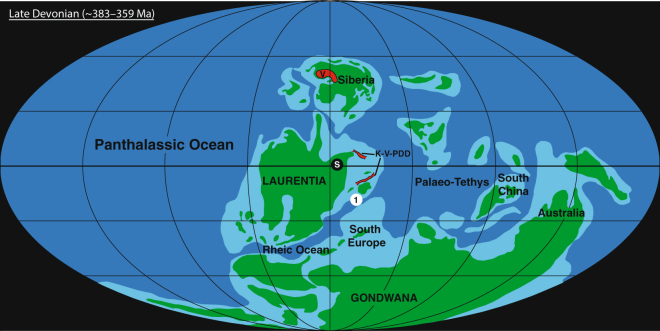
Palaeogeographic map of the Late Devonian world, based on ref.^77^. The location of Steinbruch Schmidt(1), the Siljan impact crater (S), the Viluy Traps (V), and the Kola, Vyatka, and Pripyat–Dniepr–Donets rift systems (K-V-PDD) are indicated.

The Late Devonian (∼383–359 Ma) marked a time of repeated environmental upheaval, featuring numerous carbon-cycle perturbations and local- to global-scale periods of marine anoxia during the Givetian (e.g., Frasnes Event), Frasnian (e.g., Punctata Event), and Famennian (e.g., Annulata Event) Stages. Two such episodes of environmental perturbation also featured major faunal extinctions, documented at the Frasnian–Famennian and Famennian–Tournasian Stage Boundaries (reviewed by e.g.^1,2^). The Frasnian–Famennian (FF) boundary extinction was apparently the most devastating biotic crisis of the Devonian Period, and represents one of the ‘Big Five’ mass extinctions of the Phanerozoic Eon^3,4^. Reef systems and especially stromatoporoids, which had enjoyed considerable ecological success and geographically widespread distribution throughout much of the preceding Devonian, were particularly affected (see^5^). Elevated extinction rates are also documented amongst many other benthic and planktonic marine invertebrates (reviewed by e.g.,^3,6^). The ultimate cause(s) of this extinction remains debated, with numerous potential triggers suggested including marine anoxia, extra-terrestrial impacts, and volcanic activity (Fig. 1). However, the exact age of the Frasnian–Famennian boundary remains relatively poorly constrained, hindering any comparison of the extinction date with those of impact craters or volcanic basalts. This study presents a new precise age of the FF boundary from dating of a bentonite layer deposited 2.5 m stratigraphically below that horizon, in order to better constrain temporal correlations of the FF extinction and other Late Devonian phenomena.

**Figure 2:**
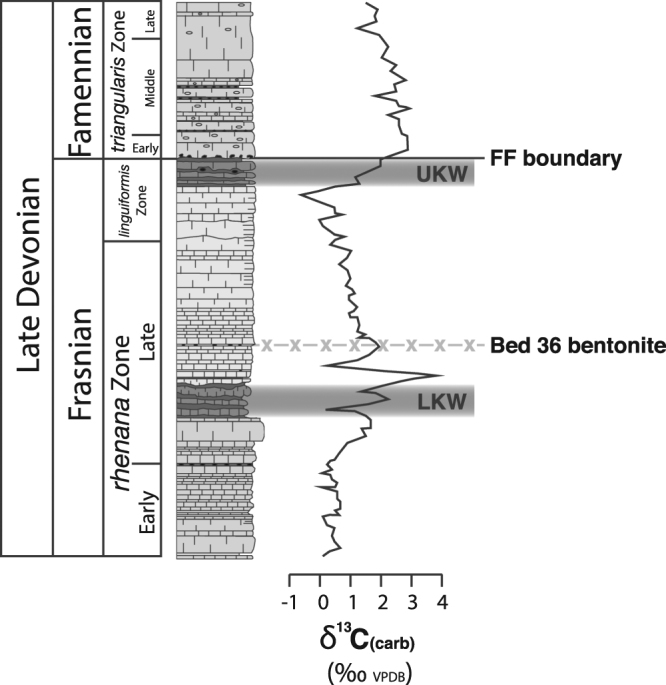
Summary stratigraphic data of the Steinbruch Schmidt section (Germany). Lithological and biostratigraphic data and the stratigraphic positions of the Kellwasser horizons are from ref.^49^. Carbon-isotope data are from ref.^7^. The stratigraphic position of the Bed 36 bentonite is from ref.^53^.

The onset of the Frasnian–Famennian extinction is documented in uppermost Frasnian strata, which also record a distinct perturbation to the carbon cycle as a positive carbon-isotope excursion. Increases in δ^13^C values of up to 4‰ in both carbonates and bulk organic matter are observed in the *linguiformis* conodont Zone in sedimentary records across the globe (e.g.,^7–12^); see also Fig. 2). An additional positive excursion in δ^13^C of carbonates and organic matter is also often documented in the preceding *rhenana* conodont Zone or its biostratigraphic equivalent. The two δ^13^C excursions stratigraphically correlate with the appearance of laminated black shales referred to as the Lower (LKW) and Upper (UKW) Kellwasser horizons, particularly in European records^7,9^; Fig. 2). The onset of decline amongst some marine fauna (such as reef systems) is thought to have begun during the LKW Event, with such trends culminating in a more severe extinction pulse affecting numerous taxanomic groups during the UKW Event, recorded at the FF boundary (reviewed in refs^1,6^). The stratigraphically correlated black shales and positive δ^13^C excursions have been used to infer the development of widespread marine anoxia during both Kellwasser events in the latest Frasnian, which is supported by pyrite-framboid size, organic-biomarker analysis, calcite sulphur-isotope compositions, and trace-metal enrichments in sedimentary records from across the globe (e.g.,^13–17^). In addition to widespread marine anoxia, there is evidence for increased marine primary productivity, enhanced rates of continental weathering, and global cooling during both of the Kellwasser events^11,17–22^. Consequently, the marine anoxic conditions during the Kellwasser events are often attributed to enhanced primary productivity stimulated by the influx of nutrients to the marine realm during times of extreme weathering rates, potentially resulting from orogenic activity and/or the expansion of rooted vascular land plants during the Late Devonian (e.g.,^23,24^).

A wide range of mechanisms has been proposed as additional external triggers of the extinction, including the impact of a large extra-terrestrial body, repeated bombardment by numerous smaller extra-terrestrial objects, large-scale volcanic activity and associated gas release, and climate forcing via orbital configuration^6,12,25–31^. Microtektite layers have been reported from lower Famennian strata of both Belgian and Chinese records; a Late Devonian impact crater is well known from Sweden (the ∼50 km diameter Siljan crater: e.g.,^32^), with another in North America indicated by an impact breccia in Upper Devonian strata of Nevada (e.g.,^33^). Late Devonian large-scale volcanic activity is best associated with the emplacement of the Viluy Traps, a Large Igneous Province (LIP) in eastern Siberia thought to have originally consisted of more than 1 Mkm^3^ of basalts^29–31,34,35^. Additional volcanic activity linked with major rifting systems in eastern Europe has also been noted as occurring during the Late Devonian, which may have generated on the order of an additional 1 Mkm^3^ of basaltic material^30,36,37^. An excellent correlation between the determined ages of LIP volcanic events and times of mass extinction and major environmental perturbation has been established for latest-Palaeozoic through to early Cenozoic times (e.g.,^35,38,39^), and the end-Cretaceous extinction has additionally been linked with a large extra-terrestrial impact (e.g.,^40^). In this context, the record of both large-scale volcanism and extra-terrestrial impacts occurring in the Late Devonian is intriguingly suggestive of a potential causal relationship between those phenomena and the many Late Devonian biotic and environmental crises, including the FF extinction.

In order to determine whether large-scale volcanism or an extra-terrestrial impact were indeed the major cause of the FF extinction, their precise temporal relationship with the extinction must be established. This approach has been employed successfully to illustrate a precise coincidence between other extinction events, such as the end-Permian and end-Triassic, and times of major intrusive/extrusive LIP magmatism (e.g.,^41–43^). However, the date of the FF extinction remains poorly constrained. The current Geological Time Scale date for the FF boundary is 372.2 ± 1.6 Ma, based on Monte Carlo statistical analysis of several Devonian ash uranium-lead (U-Pb) dates^44^. Recalculation of this timescale based on Bayesian statistics, and independent methods such as rhenium-osmium (Re-Os) isochron dating and cyclostratigraphy anchored to the well-dated Famennian–Tournasian boundary have all given a similar age, but with uncertainties still on the order of a million years^45–48^. Because major LIP magmatic events and extra-terrestrial impacts can influence the Earth’s environment on the time scale of tens of millennia or less, a much greater degree of precision is desired for the age of the FF extinction, ideally to within <100 kyr.

In this study, a bentonite layer from Bed 36 (by the numbering system of ref.^49^) in the Frasnian–Famennian succession at the abandoned Steinbruch Schmidt Quarry (Germany: Figs 1 and 2) is dated using U-Pb analysis of zircons, in order to determine a precise age of the FF boundary. Recent advances in U-Pb dating have allowed for unprecedented precision in constraining the ages of deep-time volcanic eruptions, allowing geological boundary ages to be constrained to within tens to hundreds of millennia based on dating of bentonite layers stratigraphically proximal to those boundaries (e.g.,^50–52^). The Steinbruch Schmidt bentonite layer is just 2.5 m below the FF boundary and, crucially, lies within a thin (2.5 m) set of carbonate beds stratigraphically between the two Kellwasser horizons. The time between these two horizons is estimated as 400–450 kyr^12^. Thus, the age of this bentonite represents a good approximation of the ages of the FF boundary and the Upper Kellwasser extinction event. This bentonite has previously been dated to 377.2 ± 1.7 Ma by laser ablation-inductively coupled plasma-mass spectrometry (LA-ICP-MS) techniques, from which a FF boundary age of 376.1 Ma was inferred^53^. However, the inconsistency between this date and more recent determinations of the FF boundary age based on Re-Os dating and statistical modelling of Devonian bentonite U-Pb ages (see below) suggests that this previous age might be erroneous^44–48^. Nonetheless, the previous dating by ref.^53^ highlights the suitability of the zircons in this bentonite for precise dating. In this study, the Steinbruch Schmidt bentonite is re-dated using state-of-the-art chemical abrasion-isotope dilution-thermal ionization mass spectrometry (CA-ID-TIMS) analysis with the EARTHTIME 2535 spike. Such methods allow for a very precise age of the bentonite to be determined; consequently, the FF boundary age can also be constrained to a greater level of precision. This new age is compared with the determined ages of other Late Devonian phenomena such as the Siljan impact and Viluy Trap magmatic episodes in order to establish the level of coincidence between these events and the FF extinction, and to infer if they had any influence on that biotic/environmental crisis.

## Results

**Figure 3:**
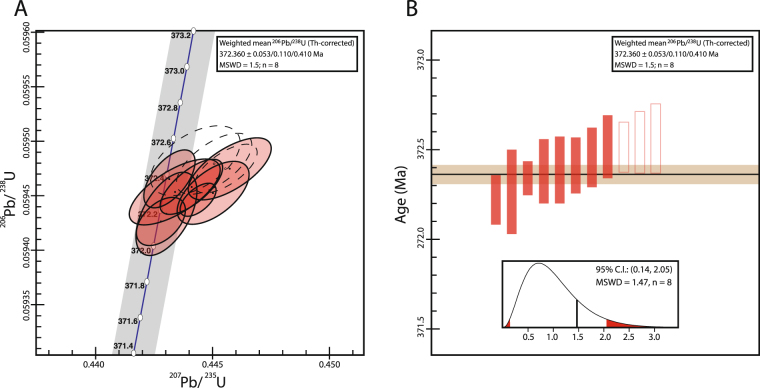
(**A**) Uranium-lead concordia diagram illustrating the compositions of the 11 analysed zircons. The ellipses indicate the 2σ uncertainties for each sample. The eight overlapping zircons used to generate a weighted mean age are shaded red with solid borders; the remaining three zircons are colourless and have dashed borders. (**B**) Illustration of the weighted mean distribution of the 8 zircons (solid red), with the additional 3 zircons also indicated (open red). The height of each bar is proportional to the 2σ uncertainty. The horizontal black line indicates the resultant age of 372.36 Ma, with the horizontal brown bar indicating the 53 kyr uncertainty in the measurement.

The zircon crystals from the bentonite chosen for U-Pb dating were all acicular euhedral long prismatic grains. 11 grains were dated by the CA-ID-TIMS technique and 8 of these produced overlapping concordant ages from which a weighted mean age of 372.360 ± 0.053/0.11/0.41 Ma (MSWD = 1.47) was calculated (see Supplementary Table 1; Fig. 3). The three uncertainties are all at 2σ, the first (0.053 Ma) represents only the measurement uncertainty, the second (0.11 Ma) represents the measurement uncertainty plus the ET2535 tracer uncertainty, and the third (0.41 Ma) includes the measurement, tracer and also decay constant uncertainties. This last, greatest, uncertainty must be employed when making comparisons between U-Pb ages and argon-argon (Ar-Ar) ages. The three zircon ages not included in the weighted mean are all slightly older. These grains may be antecrysts that crystallized somewhere in the magmatic system significantly prior to eruption.

## Discussion

**Figure 4:**
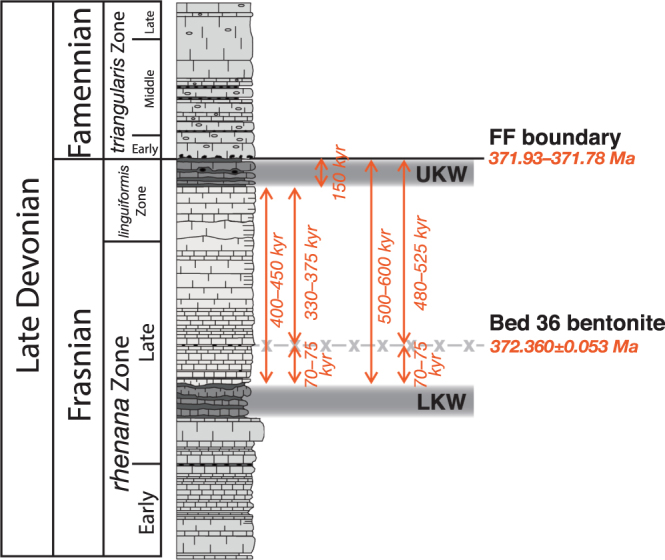
Age modelling of the Kellwasser horizons, bentonite layer, and Frasnian–Famennian boundary at Steinbruch Schmidt. Lithological and biostratigraphic data and the stratigraphic positions of the Kellwasser horizons are from ref.^49^. The stratigraphic position of the Bed 36 bentonite is from ref.^53^. Time-durations between the Kellwasser horizons and the FF boundary are from ref.^12^. Division of time between the bentonite and the Kellwasser horizons is achieved on the assumption of a constant sedimentation rate and complete sedimentary record in the carbonate layers between the Kellwasser horizons. The determined age of the Bed 36 bentonite and the inferred age of the FF boundary are from this study.

The stratigraphic position of the analysed bentonite below the FF boundary means that the age of that horizon can be no older than that of the bentonite itself. Consequently, because the bentonite has a determined age of 372.360 ± 0.053 Ma, the FF boundary cannot be older than 372.4 Ma. This result is comfortably within error of the recent estimations of the FF boundary age^44–48^ but markedly younger than the date by ref.^53^, supporting the hypothesis that the previous determined age of the Steinbruch Schmidt bentonite was erroneous. However, whilst a FF boundary age of 372.4 Ma is in agreement with other estimates for that horizon, its actual age will in reality be younger because the bentonite lies 2.5 m stratigraphically below that level. A recent study constructed a global cyclostratigraphic framework of geologic time during and between the two Kellwasser events^12^. By combining this age model with the new age of the Steinbruch Schmidt bentonite in this study, the exact age of the FF boundary can be estimated (Fig. 4). The age model in ref.^12^ indicated that the Lower Kellwasser Event terminated 500–600 kyr prior to the end of the Frasnian, with the Upper Kellwasser Event beginning 150 kyr before the conclusion of that Stage. Consequently, approximately 350–450 kyr passed between the two events, which can be further constrained to 400–450 kyr due to the recording of an entire long-eccentricity cycle (405 kyr) between the two Kellwasser horizons^12^. The Bed 36 bentonite at Steinbruch Schmidt lies 42.5 cm above the top of the Lower Kellwasser Horizon, and 206 cm below the base of the Upper Kellwasser Horizon, 17% of the stratigraphic distance between the Lower and Upper Kellwasser beds. Assuming that the age model of ref.^12^ is correct, that the record at Steinbruch Schmidt is stratigraphically complete, and the carbonate beds between the two Kellwasser horizons were deposited at a constant sedimentation rate, the bentonite would have been deposited ∼70–75 kyr after the end of the Lower Kellwasser Event, and consequently ∼480–525 kyr before the end of the Frasnian Stage. For the determined bentonite age of 372.360 ± 0.053 Ma, this reasoning results in a Frasnian–Famennian boundary age of 371.93–371.78 Ma (371.86 ± 0.08 Ma).

**Figure 5:**
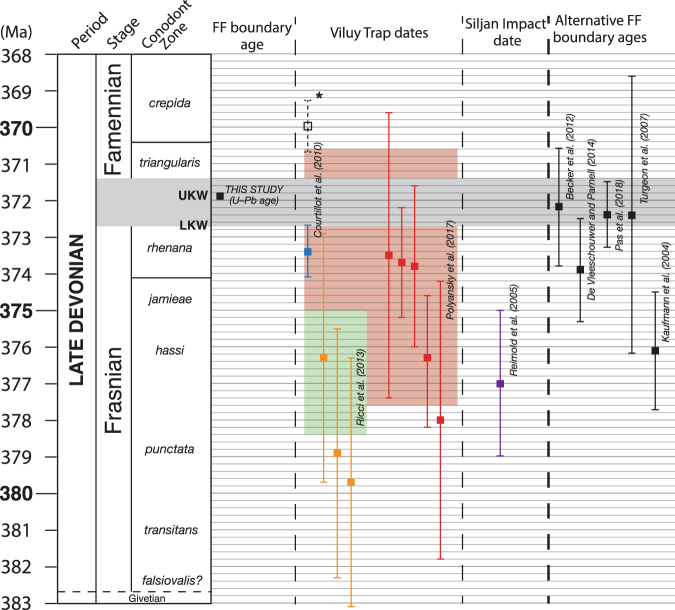
Summary diagram reviewing the ages of the FF boundary (from this work and previous studies), the Siljan impact crater, and the Viluy Traps. For the Viluy Trap dates, the illustrated dates indicate ages of individual LIP rocks. Biostratigraphy and the ages (where indicated) of biostratigraphic boundaries are based on the age model of ref.^62^. The grey bar indicates the inferred boundary age from this study after accounting for the uncertainty in calibrating between the U-Pb derived boundary age and the Ar-Ar derived ages of the Siljan impact and Viluy Trap basalts, and extends down to 372.77 Ma (the maximum age of the boundary following inclusion of the U-Pb *vs* Ar-Ar calibration uncertainty if negligible time is assumed to have passed between the deposition of the bentonite and the overlying boundary strata). Red dates are from ref.^59^, orange dates from ref.^31^. The blue Viluy Trap date is from ref.^29^ using the calibration of ref.^78^, the black open Viluy Trap date is that same Viluy Trap date from ref.^29^, using the conventional calibration. The green shaded area indicates the estimated age of the first pulse of Viluy Trap volcanism by ref.^31^, based on the dates of refs^29,31^. The red shaded area indicates the estimated age of the first pulse of Viluy Trap volcanism by ref.^59^, based on the dates of refs^29,31,59^. The FF boundary date generated in this study is also consistent with that of ref.^79^.

The current Geological Time Scale (GTS) provides an age for the base of the Famennian Stage of 372.2 ± 1.6 Ma, based on Monte Carlo statistical analysis of selected U-Pb dates from volcanic materials distributed throughout the Devonian stratigraphy^44^. This age has subsequently been refined by Bayesian statistical analyses of the same U-Pb ages, which yielded a FF boundary age of 373.9 ± 1.4 Ma^47^. Such approaches are axiomatically dependent on the accuracy and precision of the U-Pb dates utilized, many of which have error bars of >1 Myr, thus hampering the precision of the Stage boundary ages output by the model. A more direct approach is to employ the decay of ^187^Re to ^187^Os in organic-rich shale records of the FF boundary to construct a Re-Os isochron age for those sediments^45,46^. An age of 372.4 ± 3.8 Ma for the FF boundary has been calculated using this method^45^. Most recently, a cyclostratigraphic timescale was constructed for the entire Famennian based on three cores from the North American Illinois Basin^48^. By anchoring this timescale to the well constrained date of the Famennian–Tournasian boundary (358.9 ± 0.4 Ma), those authors determined an age of 372.4 ± 0.9 Ma for the FF boundary. Both the maximum and inferred FF boundary ages in this study of 372.4 Ma and 371.93–371.78 Ma, respectively, are consistent with the GTS date and the other previously determined ages documented above (Fig. 5). The inferred boundary age of 371.93–371.78 Ma is also significantly more precise than the previous estimates. Moreover, because of the stratigraphic proximity of the bentonite to the FF boundary and Kellwasser horizons at Steinbruch Schmidt, cyclostratigraphic analysis of the sediments across that single record will enable the ages of both the boundary and the Kellwasser events to be constrained still more precisely and without the assumptions inherent in the date presented here. However, even without such further investigative analyses, the temporal relationships between Late Devonian volcanic and impact events and the FF boundary extinction can still be reviewed in light of the new date of the Steinbruch Schmidt bentonite, and the inferred FF boundary age of 371.93–371.78 Ma.

The most likely candidate for an extra-terrestrial impact associated with the Late Devonian extinctions is documented by the Siljan crater in Sweden. Precise dating of the Siljan crater has been substantially hindered by significant alteration of many of the crater lithologies^32^. The most recently determined, and precise, Ar-Ar date of the crater is 377 ± 2 Ma^32^. Even accounting for the large degree of uncertainty in this age, the results of this study confirm previous findings that the FF boundary extinction significantly post-dated the impact that formed the Siljan crater (Fig. 5). Additionally, the Siljan ring has only a ∼50 km diameter, and whilst claims have been made that the crater may well have been somewhat larger originally, even the largest estimate is well under 100 km^54^. Thus, the Siljan impactor would have been only 30–50% of the size of the Chicxulub impactor linked with the end-Cretaceous extinction^40^ as well as being significantly smaller than other impactors not currently associated with biospheric crises (e.g., Manicouagan, Canada: Late Triassic).

The Alamo impact breccia in Nevada (USA) provides evidence for a further impact during the Late Devonian, but has been biostratigraphically dated to the *punctata* conodont Zone from the early Frasnian^33^. Consequently, that impact would also have substantially predated the Kellwasser events. Microtektite glasses have been reported from lower Famennian sediments in South China and Belgium as evidence for an impact^25–28^ but they are not known from the majority of FF boundary records worldwide. There is also little evidence for an impact-related iridium anomaly in FF boundary strata^55–57^. Moreover, even if an impact did occur to produce the microtektite beds, its temporal relationship with the FF extinction is unclear, with some microtektite deposits appearing above the FF boundary and others below that horizon^25–28^. This lack of stratigraphic and geographic continuity in the microtektite deposits clearly does not support a coincidence between a large-scale impact and the FF boundary extinction, although an alternative scenario of repeated bombardment by smaller extra-terrestrial objects has also been proposed (see ref.^6^).

A potential link between volcanic activity and the FF extinction has long been mooted (e.g.,^58^), but has become increasingly popular in recent years due both to dating of Viluy Trap basalts as Late Devonian in age^29,31,59^ and the clear relationship between LIP volcanism and many Mesozoic episodes of environmental perturbation and extinction (e.g.,^38,39^). Because much of the province has been eroded, the true volume of the Viluy Traps is not well constrained, but it is estimated to have originally consisted of 0.3–1 Mkm^3^ of igneous material^31,60^ the larger volume of which is comparable to that of several Phanerozoic LIPs associated with episodes of mass extinction and/or environmental change^2,61^. Some intrusive and extrusive magmas have been dated using Ar-Ar chronology, and indicate at least two pulses of Viluy magmatism, one in the late Frasnian, and another in the late Famennian^29,31,59^. The first, late Frasnian, magmatic pulse was dated to 376.7 ± 1.7 Ma by ref.^31^ and 374.1 ± 3.5 Ma by ref.^59^. The latter age does overlap with the FF boundary, although only because of the large error bars in the inferred age of that magmatic pulse. The given ages of individual basalts do not coincide with the FF boundary even once uncertainties in comparing the new U-Pb derived boundary age with Ar-Ar ages of volcanics are accounted for, although some may be closer in age to the Lower Kellwasser Event (∼372.6 Ma: Fig. 5). Consequently, if faunal extinctions did indeed commence during that earlier event, this may support a role for volcanism in the FF extinctions, even though there is no direct correlation between the ages of volcanism and the main extinction pulse at the FF boundary. The large uncertainties in the dates of individual basalts do overlap with the age of the FF boundary (Fig. 5); because these uncertainties are so large, more precise dating of Viluy Trap rocks are required in order to determine whether the FF boundary extinction truly coincided with major igneous activity. Interestingly, the second pulse of Viluy Trap volcanism (364.4 ± 1.7 Ma by ref.^31^ and 363.7 ± 0.7 Ma by ref.^59^) does match the age of the Fammenian Annulata Event based on the late Famennian cyclostratigraphic timescale of ref.^62^ (see Supplementary Figure 1).

In addition to Viluy Trap volcanism, substantial Late Devonian volcanic activity is thought to have occurred along the Kola, Vyatka, and Pripyat–Dniepr–Donets rift systems in what is now Eastern Europe (reviewed in ref.^30^). However, volcanic activity on these rifting margins likely represented a somewhat different style of volcanism to a Mesozoic LIP, and their impact on the Devonian palaeoenvironment may not be directly comparable with that of the large-scale volcanic episodes associated with Mesozoic extinction/climate events. Dating of the volcanism associated with these rift systems is also very poorly constrained: the Pripyat–Dniepr–Donets rifting is modelled as chiefly Famennian in age^37^ whilst the Kola volcanics are dated to the mid-Frasnian^63^. On the basis of current evidence, it appears unlikely that these rifts were the sole direct cause of the FF boundary extinction, although they may have played a contributing role in the build-up to one or more of the Late Devonian crises.

Whilst currently identified and dated episodes of large-scale volcanic activity do not appear to have clearly coincided with the Kellwasser events or the main pulse of extinction recorded at the FF boundary, such volcanism was clearly a feature of the Frasnian and Famennian Stages. It cannot be discounted that large-scale volcanic activity did occur coincident with the FF boundary extinction, but that the igneous products have not yet been identified or dated, or may not have been preserved. The application of indirect proxies of large-scale volcanism, such as sedimentary mercury concentrations (e.g.^64–67^) and osmium-isotopes (e.g.^68,69^), on strata from the Kellwasser horizons may give indirect evidence of the existence or absence of major volcanic activity at that time. A recent study of three FF boundary records shows mercury enrichments occurring at or near the boundary, indicating that volcanic activity may indeed have been occurring during the Upper Kellwasser Event^70^. Clearly, further work is needed to generate (precise) dates of more basalts from both the Viluy Traps and the Kola, Vyatka, and Pripyat–Dniepr–Donets rift-systems in order to verify the temporal relationship between large-scale volcanism and the Frasnian–Famennian extinction.

## Conclusions

Precisely constraining the timing of the Frasnian–Famennian (FF) mass extinction represents a crucial step in understanding the influence of external phenomena such as large-scale volcanism and meteor impacts in causing that event. This study presents a new precise age of 372.360 ± 0.053 Ma for a bentonite stratigraphically proximal to the FF boundary, confirming an age no older than 372.4 Ma for that horizon, with an actual boundary age of 371.93–371.78 Ma reconstructed on the basis of a published age model of the Frasnian–Famennian transition. This result is consistent with other recently reported ages for the Frasnian–Famennian boundary, but is considerably more precise than these previously published works. The date also creates an anchor point for use in future cyclostratigraphic models which could establish an even more precise age for the FF boundary and associated Kellwasser events. Importantly, a greater confidence in the precise age of this horizon allows for a better understanding of the temporal relationships between the extinction event that took place at that time, and phenomena that might have contributed to the extinction such as extra-terrestrial impacts and large-scale volcanism. It is confirmed that the Siljan impact event happened significantly prior to the FF extinction, and is therefore unlikely to have played a role in that event. No individual Viluy Trap basalt matches the FF boundary date, although the timing of that horizon does fall within the age uncertainty of a late Frasnian pulse in Viluy Trap volcanism, and some individual basalts are close in age to the Lower Kellwasser Event. Thus, although there is no direct evidence of a coincidence between the extinction and large-scale volcanism based on current geochronology, major volcanic activity was likely still prevalent during the late Frasnian, highlighting the need for further geochronological and chemostratigraphic work to confirm the existence or absence of major volcanic activity during the Frasnian–Famennian extinction.

## Materials and Methods

∼1 kg of bentonite material was sampled from the Bed 36 layer (by the numbering of ref.^49^) at Steinbruch Schmidt, ∼1 km north of Braunau, near the town of Bad Wildungen, Hesse, Germany (51° 5′ 12.1′′ N, 9° 7′ 53.9′′ E). Zircon crystals from this bentonite were separated by conventional mineral separation techniques and analysed by chemical abrasion-isotope dilution-thermal ionization mass spectrometry (CA-ID-TIMS) at the Département des sciences de la Terre, Université de Genève following the techniques described in ref.^43^. The bentonite contained abundant zircon crystals in many shapes and sizes, however only euhedral acicular elongated grains were selected for dating since these are least likely to be re-worked grains. 15 of the most pristine crystals were selected and annealed at 900 °C for 48 hr. The crystals were then individually cleaned with 3 N molar HNO_3_ and loaded into 200 μl Savillex microcapsules for chemical abrasion to remove any areas of the grains that may have experienced Pb loss. Chemical abrasion was conducted in 3 drops of concentrated HF at 210 °C for 12 hr following the recommendation of ref.^71^. Eleven of the 15 grains survived the chemical abrasion procedure, these grains were then cleaned in 6 molar HCl in 3 ml Savillex beakers at 80 °C for 24 hr and then ultrasonically cleaned in 3 molar HNO_3_. Following cleaning, the grains were loaded into pre-cleaned 200 μl Savillex microcapsules with 3 drops of concentrated HF and trace HNO_3_ and one drop of the ET ^202^Pb + ^205^Pb + ^233^U + ^235^U tracer solution^72,73^. The microcapsules were placed in a Parr bomb, and then into an oven at 210 °C for 48 hr for dissolution. The resultant solution was dried down and the residue converted to a chloride by reaction with concentrated HCl at 210 °C for 12 hr, before being added to columns filled with pre-cleaned anion exchange resin. The Pb and U cuts were collected from the columns and dried down in cleaned 7 ml Savillex beakers with trace H_3_PO_4_ before being loaded on to outgassed zone refined Re filaments with a Si-Gel emitter and placed into a Phoenix Isotopx TIMS mass spectrometer. Pb was measured in dynamic mode on a daly; the U was measured as an oxide in static mode on faraday cups equipped with 10^12 Ω resistors. Mass fractionation of Pb was corrected using a known ^202^Pb/^205^Pb of 0.99924; for U, ^233^U/^235^U of 0.99506 was used along with a sample ^238^U/^235^U of 137.818 ± 0.045 (2σ^74^). All data was processed using tripol and redux software packages, which utilise the algorithms of ref.^75^. All ages are corrected for initial ^230^Th disequilibria assuming a partition coefficient relationship Th_(zircon/rock)_/U_(zircon/rock)_ of 0.2^76^ with the correction for disequilibria resulting in a ~90 kyr increase in the age of each grain. All data along with the long-term isotopic composition of the blank from the UNIGE lab used in this study are reported in Supplementary Table 1.

## Electronic supplementary material

Supplementary information
