# Supplementary material for: Precisely dating the Frasnian–Famennian boundary: implications for the cause of the Late Devonian mass extinction
Source: Sci Rep. 2018 Jun 22;8:9578. doi: 10.1038/s41598-018-27847-7 (PMC6014997; doi:10.1038/s41598-018-27847-7)
Supplement: Supplementary file 1 — Supplementary information [file 41598_2018_27847_MOESM1_ESM.pdf]

Precisely dating the Frasnian–Famennian boundary: implications for the cause of the Late Devonian mass extinction – Supplementary Information

L.M.E. Percival[1*], J.H.F.L. Davies[2], U. Schaltegger[2], D. De Vleeschouwer[3], A.-C. Da Silva[4,5], K.B. Föllmi[1]

1: Institut des sciences de la Terre, Géopolis, Université de Lausanne, 1015 Lausanne, Switzerland

2: Département des sciences de la Terre, Université de Genève, 1205 Genève, Switzerland

3: MARUM—Center for Marine Environmental Sciences, University of Bremen, Leobenerstraße, 28359 Bremen, Germany

4: Sedimentary Petrology Laboratory, Liège University, Sart Tilman B20, Allée du Six Août 12, 4000 Liège, Belgium

5: Paleomagnetic Laboratory, Utrecht University, Budapestlaan 17, 3584 CD Utrecht, The Netherlands

**Corresponding author: lawrence.percival@unil.ch*

Supplementary Table 1: U-Pb analytical data

Dev-Ben

	Composition					Dates (Ma)										Isotopic Ratios						
	Th/U *a*	Pb* (pg) *b*	Pb c (pg) *d*	Pb*/Pb c *e*	Th/U (magma) *f*	$^{206}Pb/^{238}U$ *g*	±2σ abs	$^{206}Pb/^{238}U$ <Th> *h*	±2σ abs	$^{207}Pb/^{235}U$ *g*	±2σ abs	$^{207}Pb/^{206}Pb$ *g*	±2σ abs	Corr. coef.	% disc. *i*	$^{206}Pb/^{204}Pb$ *j*	$^{206}Pb/^{238}U$ *k*	±2σ %	$^{207}Pb/^{235}U$ *k*	±2σ %	$^{207}Pb/^{206}Pb$ *k*	±2σ %
Dev-Ben_z1	0.33	19.0	0.44	43	1.650	372.257	0.091	372.339	0.094	373.34	0.53	380.1	3.6	0.563	2.06	2599	0.059446	0.025	0.44439	0.17	0.054241	0.16
Dev-Ben_z2	0.61	15.4	0.43	36	3.050	372.14	0.13	372.22	0.14	372.22	0.74	372.7	5.0	0.473	0.16	2018	0.059426	0.037	0.4428	0.24	0.05406	0.22
Dev-Ben_z3	0.54	9.73	0.35	28	2.700	372.33	0.15	372.41	0.15	373.14	0.94	378.2	6.1	0.692	1.55	1586	0.059458	0.043	0.4441	0.30	0.05420	0.27
Dev-Ben_z4	0.24	12.1	0.37	33	1.200	372.37	0.16	372.46	0.16	372.47	0.77	373.1	5.0	0.594	0.19	2062	0.059465	0.045	0.4432	0.25	0.05407	0.22
Dev-Ben_z5	0.79	16.5	0.66	25	3.950	372.30	0.18	372.38	0.18	373.8	1.1	383.0	7.1	0.582	2.80	1356	0.059453	0.049	0.4450	0.34	0.05431	0.32
Dev-Ben_z7	1.04	9.19	0.37	25	5.200	372.46	0.17	372.54	0.17	373.8	1.1	382.3	7.5	0.623	2.57	1276	0.059479	0.047	0.4451	0.36	0.05429	0.33
Dev-Ben_z8	0.28	10.5	0.67	16	1.400	372.30	0.18	372.38	0.19	372.6	1.4	374.8	9.1	0.673	0.66	981	0.059453	0.051	0.4434	0.44	0.05411	0.40
Dev-Ben_z9	0.46	12.9	0.52	25	2.300	372.43	0.14	372.51	0.14	374.19	0.95	384.9	6.3	0.672	3.23	1468	0.059475	0.038	0.4456	0.30	0.05436	0.28
Dev-Ben_z11	0.58	13.0	0.73	18	2.900	372.48	0.19	372.56	0.19	373.3	1.3	378.1	9.1	0.518	1.49	1022	0.059482	0.053	0.4443	0.43	0.05419	0.40
Dev-Ben_z12	0.61	12.2	0.62	20	3.050	372.43	0.17	372.51	0.17	374.3	1.2	385.8	8.3	0.657	3.47	1114	0.059475	0.048	0.4457	0.40	0.05438	0.37
Dev-Ben_z13	0.49	9.22	0.25	36	2.450	372.18	0.23	372.26	0.23	372.39	0.91	373.7	5.9	0.546	0.40	2111	0.059433	0.064	0.4430	0.29	0.05409	0.26

a: Th contents calculated from radiogenic ^{208}Pb and ^{230}Th-corrected ^{206}Pb/^{238}U date of the sample, assuming concordance between U-Pb and Th-Pb systems.
b: Total mass of radiogenic Pb.
d: Total mass of common Pb.
e: Ratio of radiogenic Pb (including ^{208}Pb) to common Pb.
f: Th/U ratio of magma from which mineral crystallised.
g: Isotopic dates calculated using λ238 = 1.55125E-10 (Jaffery *et al.*, 1971) and λ235 = 9.8485E-10 (Jaffery *et al.*, 1971).
h: Corrected for initial Th/U disequilibrium using radiogenic ^{208}Pb and Th/U_{magma} specified.
i: % discordance = 100 – (100x(^{206}Pb/^{238}U date)/(^{207}Pb/^{206}Pb date)).
j: Measured ratio corrected for fractionation and spike contribution only.
k: Measured ratios corrected for fractionation, tracer, and blank.

NB: Red data indicate the three zircons grains with older ages that were not included in the weighted mean.

Supplementary Figure 1: Late Devonian age models and Viluy Trap volcanism

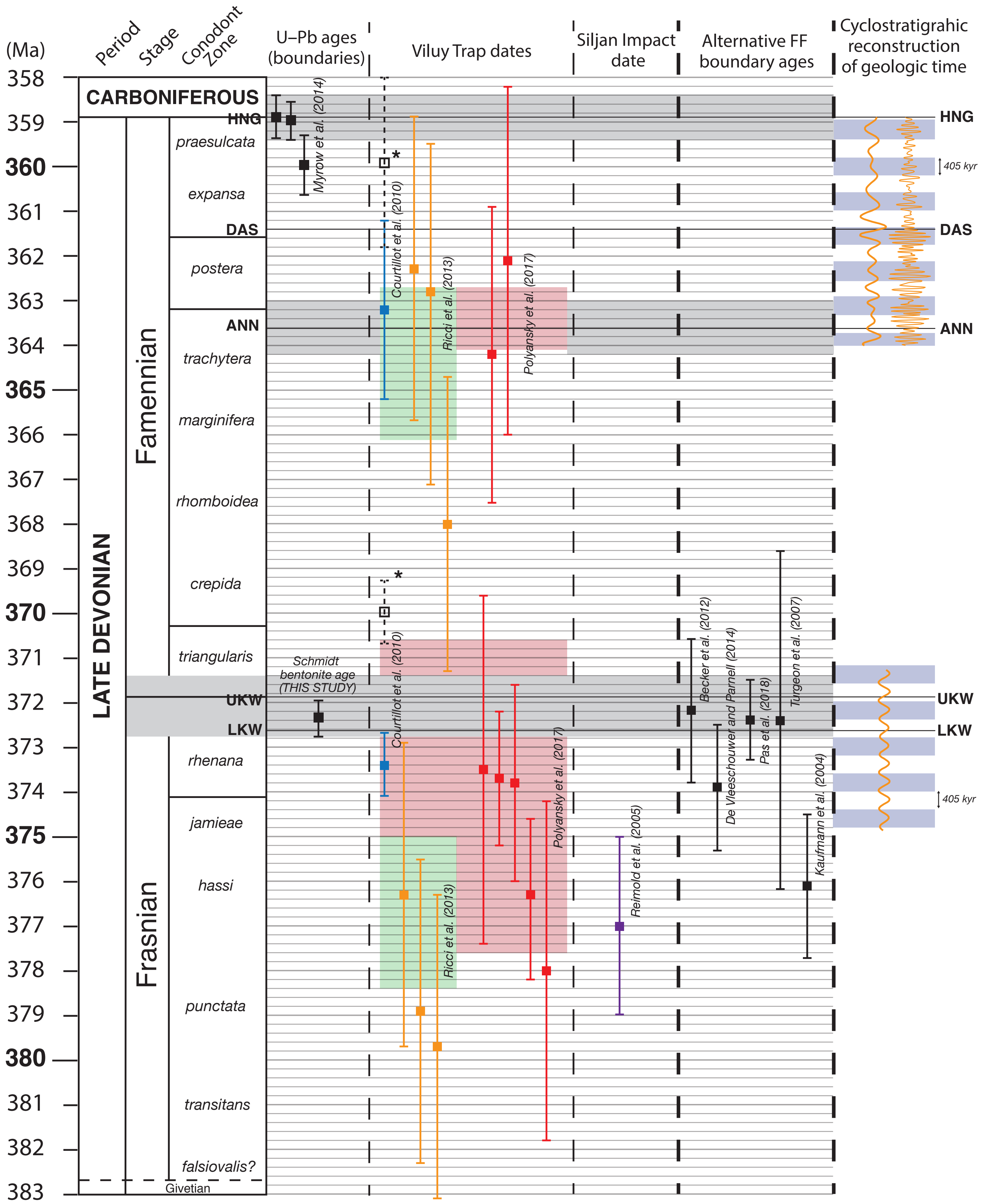

Summary diagram of Late Devonian geologic time, with ages for the FF and Devonian–Carboniferous boundaries (from this and other studies), the Siljan impact crater, and the Viluy Traps. FF boundary cyclostratigraphy is from De Vleeschouwer *et al*. (2017), and is anchored to the bentonite age of 372.36±0.410 Ma (*this study*). Upper Famennian cyclostratigraphy is from De Vleeschouwer *et al*. (2013) and is anchored to the Hangenberg horizon age of 358.93±0.45 Ma (Myrow *et al*., 2014), to give an age of ~363.6 Ma for the Annulata event, coincident with the second pulse of Viluy Trap volcanism. For the Viluy Trap dates, the illustrated dates indicate ages of individual LIP rocks. The grey bars indicate ages of the FF boundary, Annulata, and Hangenberg, based on U–Pb ash dates (anchored to cyclostratigraphic age model for the Annulata) accounting for comparing calibrations in the U–Pb and Ar–Ar systems. Red dates are from Polyansky *et al*. (2017), orange dates from Ricci *et al*. (2013). The blue Viluy Trap date is from Courtillot *et al*. (2010) using the calibration of Renne et al. (2010), the black open Viluy Trap date is that same Viluy Trap date from Courtillot *et al*. (2010), using the conventional calibration. Green and red shaded areas indicate the estimated ages of the two pulses of Viluy Trap volcanism based on the works of Ricci *et al*. (2013) and Polyansky *et al*. (2017), respectively.

SUPPLEMENTARY INFORMATION REFERENCES:

Becker, R.T., Gradstein, F.M. and Hammer, Ø., 2012, The Devonian Period. In: The Geological Time Scale, 2012, p. 559–601.

Courtillot, V., Kravchinsky, V.A., Quidelleur, X., Renne, P.R. and Gladkochub, D.P., 2010, Preliminary dating of the Viluy traps (Eastern Siberia): Eruption at the time of Late Devonian extinction events? Earth and Planetary Science Letters, 300, p. 239–245, doi:10.1016/j.epsl.2010.09.045.

De Vleeschouwer, D. and Parnell, A.C., 2014, Reducing time-scale uncertainty for the Devonian by integrating astrochronology and Bayesian statistics. Geology, 42, p. 491-494, doi:10.1130/G35618.1.

De Vleeschouwer, D., Rakociński, M., Racki, G., Bond, D.P., Sobień, K. and Claeys, P., 2013, The astronomical rhythm of Late-Devonian climate change (Kowala section, Holy Cross Mountains, Poland). Earth and Planetary Science Letters, 365, p. 25–37, doi:10.1016/j.epsl.2013.01.016.

De Vleeschouwer, D., Da Silva, A.C., Sinnesael, M., Chen, D., Day, J.E., Whalen, M.T., Guo, Z. and Claeys, P., 2017, Timing and pacing of the Late Devonian mass extinction event regulated by eccentricity and obliquity. Nature communications, 8, doi:10.1038/s41467-017-02407-1.

Jaffey, A.H., Flynn, K.F., Glendenin, L.E., Bentley, W.T. and Essling, A.M., 1971, Precision measurement of half-lives and specific activities of ^{235}U and ^{238}U. Physical Review C, p. 1889, doi:10.1103/PhysRevC.4.1889.

Kaufmann, B., Trapp, E. and Mezger, K., 2004, The numerical age of the upper Frasnian (Upper Devonian) Kellwasser horizons: A new U-Pb zircon date from Steinbruch Schmidt (Kellerwald, Germany). The Journal of geology, 112, p. 495–501, doi:10.1086/421077.

Myrow, P.M., Ramezani, J., Hanson, A.E., Bowring, S.A., Racki, G. and Rakociński, M., 2014, High-precision U–Pb age and duration of the latest Devonian (Famennian) Hangenberg event, and its implications. Terra Nova, 26, p. 222–229, doi:10.1111/ter.12090.

Pas, D., Hinnov, L., Day, J.E.J., Kodama, K., Sinnesael, M. and Liu, W., 2018, Cyclostratigraphic calibration of the Famennian stage (Late Devonian, Illinois Basin, USA). Earth and Planetary Science Letters, 488, p. 102-114, doi:10.1016/j.epsl.2018.02.010.

Polyansky, O.P., Prokopiev, A.V., Koroleva, O.V., Tomshin, M.D., Reverdatto, V.V., Selyatitsky, A.Y., Travin, A.V. and Vasiliev, D.A., 2017, Temporal correlation between dyke swarms and crustal extension in the middle Palaeozoic Vilyui rift basin, Siberian platform. Lithos, 282, p. 45–64, doi:10.1016/j.lithos.2017.02.020.

Reimold, W.U., Kelley, S.P., Sherlock, S.C., Henkel, H. and Koeberl, C., 2005, Laser argon dating of melt breccias from the Siljan impact structure, Sweden: Implications for a possible relationship to Late Devonian extinction events. Meteoritics & Planetary Science, 40, p. 591–607, doi:10.1111/j.1945-5100.2005.tb00965.x.

Renne, P.R., Mundil, R., Balco, G., Min, K. and Ludwig, K.R., 2010, Joint determination of 40K decay constants and 40Ar*/40K for the Fish Canyon sanidine standard, and improved accuracy for 40Ar/39Ar geochronology. Geochimica et Cosmochimica Acta, 74, p. 5349–5367, doi:10.1016/j.gca.2010.06.017.

Ricci, J., Quidelleur, X., Pavlov, V., Orlov, S., Shatsillo, A. and Courtillot, V., 2013, New 40Ar/39Ar and K–Ar ages of the Viluy traps (Eastern Siberia): further evidence for a relationship with the Frasnian–Famennian mass extinction. Palaeogeography, Palaeoclimatology, Palaeoecology, 386, p. 531–540, doi:10.1016/j.palaeo.2013.06.020.

Turgeon, S.C., Creaser, R.A. and Algeo, T.J., 2007, Re–Os depositional ages and seawater Os estimates for the Frasnian–Famennian boundary: implications for weathering rates, land plant evolution, and extinction mechanisms. Earth and Planetary Science Letters, 261, p. 649–661, doi:10.1016/j.epsl.2007.07.031.
